# From Competence to Mastery: Reframing Neurology's Approach to Competency-Based Education

**DOI:** 10.1212/NE9.0000000000200300

**Published:** 2026-02-25

**Authors:** Roy Strowd

**Affiliations:** From the Department of Neurology, Wake Forest University School of Medicine, Winston Salem, NC.

This issue of *Neurology*®* Education* arrives at a moment when neurology is actively debating the future of competency-based medical education (CBME). Alongside studies on entrustable professional activities (EPAs) for advanced practice providers (APPs) and simulation-based assessment, this issue spans contemporary educational needs from academic half-day redesign^1^ to antiracism training,^2^ fellowship development in epilepsy^3^ and movement disorders,^4^ transcranial Doppler (TCD) simulator-based training,^5^ artificial intelligence (AI) education gaps,^6^ and neurologic-emergency curricula for first responders.^7^ Across these diverse learner groups and contexts, the articles illustrate both the promise and the complexity of embracing competency-informed pathways: using time intentionally, clarifying the developmental arc from novice to competence to mastery, and ensuring that our assessments measure the authentic work that clinicians in neurology must be trusted to perform.

## Why Competence Is Not Enough: Trust as a Component of Readiness for Practice

In their Viewpoint, Patino et al.^8^ highlight a central limitation of milestone-only assessment: it may document component skills without fully capturing a trainee's ability to integrate competencies to deliver safe, unsupervised care. Their call to develop national EPAs for neurology residency underscores a perceived gap. EPAs describe the integrated, observable work clinicians must be entrusted to perform (i.e., localizing lesions, initiating emergent evaluation, interpreting data, managing uncertainty, and communicating across teams). Milestones often document decisions that have already been made. EPAs rest on a foundation of earned trust where supervising faculty do not just judge knowledge or technical skill, they determine whether a trainee can be safely given responsibility.^9^

## Expanding EPAs Across the Neurology Workforce

Two additional articles extend the EPA framework into APP training, building important infrastructure for workforce readiness for the field broadly.

Harrison et al.^10^ present consensus EPAs for general neurology APPs, developed through a modified Delphi process. These EPAs offer a structured framework for onboarding APPs whose previous neurologic exposure varies widely, clarifying expectations for supervision and articulating standards for safe early practice.

Their accompanying study evaluating the relationship between supervisor-assessed entrustment and performance in simulated emergencies offers valuable validity evidence.^11^ EPA-based faculty ratings correlated with checklist-based performance. Learner self-assessment showed substantial alignment with supervisor judgment. Together, these findings demonstrate that EPAs can serve not only as curricular scaffolds but also as credible, performance-linked assessment tools that go beyond a skill checklist and provide a window into ability, reliability, integrity, and appropriate help-seeking behavior.

## Competence, Confidence, and the Challenge of Measuring What Matters

Confidence remains a frequent end point in neurology education research. While confidence matters, as learners must act on what they know, it is an imperfect surrogate for readiness. Confidence may actually be highest early in training, when learners have not yet encountered the complexity and variability that characterize real clinical practice. As experience grows, confidence can dip, reflecting a humility that signals deeper understanding rather than deficiency. This calibration is a hallmark of development toward mastery.

EPA-based self-assessment offers a more rigorous alternative. When learners judge themselves using entrustment anchors rather than general confidence ratings, their self-evaluation can be tied to specific work and expected outcomes. In this issue, observed alignment between learner self-assessment and faculty entrustment supports this approach. Humility is a key component of entrustability in CBME. Supervisors grant autonomy not only based on clinical ability but on a learner's recognition of their limits, transparency about uncertainty, and willingness to seek help appropriately. These trainees are entrusted with responsibility earlier than those who project confidence without insight.

## Making Time Count: Simulation as Deliberate Practice for High-Stakes Skills

The simulator-based TCD training article provides a clear example of how CBME can leverage simulation to accelerate early skill development and entrustment.^5^ TCD is a critical neuroimaging modality with evidence-based indications across cerebrovascular care, yet its use remains limited by a shortage of trained practitioners. The authors evaluate a simulator curriculum integrating real-time feedback, deliberate practice, and high-fidelity Doppler modeling.

As noted, post-test performance (61% correct insonation; 64% knowledge) “does not represent mastery or even competence” but reflects a strong foundation on which competence can be built. This is exactly where simulation aligns with CBME: cultivating foundational capability, allowing repeated practice without patient risk, and enabling standardized assessment of technical tasks. However, simulation cannot be the terminal assessment. Advancing to clinical competence and to the entrustment required for indirect supervision or independent practice requires longitudinal development in real environments where competence, reliability, and integrity can all be assessed. Clinicians need to apply skills across varied patients; demonstrate consistent performance; exercise judgment and humility; collaborate within teams; and navigate uncertainty. Simulation can build and assess the on-ramp, but entrustment must be earned in practice.

## From Novice to Mastery: A Developmental Trajectory for Neurology Training

Competency frameworks help educators articulate a developmental progression:

1. *Novice*—applying rules, needing close supervision
2. *Developing*—integrating skills inconsistently, building pattern recognition
3. *Competent*—performing safely with indirect supervision
4. *Proficient*—anticipating, adapting, synthesizing across contexts
5. *Mastery*—demonstrating expert judgment, nuance, and leadership

This work is not new. Neurology has long been committed to developing lifelong learners. CBME and EPA-based terminology do not replace that tradition; they offer ways to speak to and assess the developmental trajectory learners already travel. EPAs define competence at a work-based threshold of entrustment. Deliberate use of time and experience then propels clinicians beyond competence toward proficiency and mastery. EPAs should be interpreted as structured documentation of professional trust over time, best informed by repeated observations and narrative assessments.

## A Call for a Balanced Future: Competency-Informed, Time-Respected, Performance-Anchored Training Centered Around Trust

Taken together, the articles in this issue point toward a balanced future for neurology education, one that

1. Defines competence through entrustable clinical work
2. Links entrustment to performance in authentic contexts
3. Elevates trust as an essential part of the learner-faculty dialogue
4. Uses simulation to make time more educationally efficient
5. Treats confidence as meaningful only when calibrated
6. Recognizes time, mentorship, and experience as essential to mastery

Ultimately, what matters is whether our assessments meaningfully shape how learning occurs. New technologies now offer insight into the clinical experiences learners actually encounter and how they interpret and reflect on those moments. While we cannot control which patients they see, we can help them derive the right learning from those encounters through timely feedback, targeted simulation, and intentional workplace-based reflection and assessment. In this way, assessment becomes more than a checklist of skill attainment; it helps shape the trajectory from foundational capability toward competence, proficiency, and the mastery that neurology demands.

This is a great issue of *Neurology*®* Education*; trust me. Enjoy your reading.
